# The Genitourinary Syndrome of Menopause: An Overview of the Recent Data

**DOI:** 10.7759/cureus.7586

**Published:** 2020-04-08

**Authors:** Kyveli Angelou, Themos Grigoriadis, Michail Diakosavvas, Dimitris Zacharakis, Stavros Athanasiou

**Affiliations:** 1 Urogynecology Unit, Alexandra Hospital-National and Kapodistrian University of Athens, Athens, GRC

**Keywords:** genitourinary syndrome of menopause, vaginal atrophy, vaginal dryness, dyspareunia, estrogen replacement therapy, laser therapy

## Abstract

The genitourinary syndrome of menopause (GSM) is a relatively new term for the condition previously known as vulvovaginal atrophy, atrophic vaginitis, or urogenital atrophy. The term was first introduced in 2014. GSM is a chronic, progressive, vulvovaginal, sexual, and lower urinary tract condition characterized by a broad spectrum of signs and symptoms. Most of these symptoms can be attributed to the lack of estrogen that characterizes menopause. Even though the condition mainly affects postmenopausal women, it is seen in many premenopausal women as well. The hypoestrogenic state results in hormonal and anatomical changes in the genitourinary tract, with vaginal dryness, dyspareunia, and reduced lubrication being the most prevalent and bothersome symptoms. These can have a great impact on the quality of life (QOL) of the affected women, especially those who are sexually active. The primary goal of the treatment of GSM is to achieve the relief of symptoms. First-line treatment consists of non-hormonal therapies such as lubricants and moisturizers, while hormonal therapy with local estrogen products is generally considered the “gold standard’’. Newer therapeutic approaches with selective estrogen receptor modulators (SERMs) or laser technologies can be employed as alternative options, but further research is required to investigate the viability and scope of their implementation in day-to-day clinical practice.

## Introduction and background

The genitourinary syndrome of menopause (GSM) is a relatively new term, first introduced in 2014 by a consensus of the International Society for the Study of Women's Sexual Health and the North American Menopause Society. GSM, previously known as vulvovaginal atrophy, atrophic vaginitis, or urogenital atrophy, is a term that describes the spectrum of changes caused by the lack of estrogens during menopause [1]. GSM-like symptoms may also be present in 15% of premenopausal women due to the hypoestrogenic state [2]. Nonetheless, the vast majority of women suffering from GSM are of older age, with 50-70% of postmenopausal women being symptomatic at least to some degree [3]. To this day, GSM remains extremely underdiagnosed despite its high prevalence, mostly because of the reluctance among women to seek help due to embarrassment, or as a result of a tendency among many women to consider it as a normal feature of natural aging. However, in many cases, the reluctance of healthcare professionals to address these issues constitutes a major cause of the lack of awareness about this syndrome among affected women [4,5].

## Review

Clinical manifestations and evaluation 

GSM is a chronic, progressive condition of the vulvovaginal and lower urinary tract, which is characterized by a broad spectrum of signs and symptoms. The common clinical manifestations of the condition are summarized in Table 1.

**Table 1:** Major clinical manifestations of GSM GSM: genitourinary syndrome of menopause

Signs and symptoms of GSM	
Genital	Vaginal dryness
	Irritation/burning/itching
	Leukorrhea
	Thinning/graying pubic hair
	Vaginal/pelvic pain and pressure
	Vaginal vault prolapse
Sexual	Dyspareunia
	Reduced lubrication
	Post-coital bleeding
	Decreased arousal, orgasm, desire
	Loss of libido, arousal
	Dysorgasmia
Urinary	Dysuria
	Urgency
	Stress/urgency incontinence
	Recurrent urinary tract infections
	Urethral prolapse
	Ischemia of vesical trigone

The diagnosis of GSM may prove to be challenging as the clinical manifestations of GSM are mild and nonspecific in approximately 50% of postmenopausal women [2]. An observational study by Moral et al. found that vaginal dryness is the most prevalent and bothersome symptom as it affects up to 93% of women; the study also noted that this symptom is characterized as being moderate to severe in intensity in 68% of the cases [3]. Irritation and burning/itching of vulva/vagina are other symptoms that women with GSM frequently complain about, and they are reported in 63.3% of the affected women. The most predominant complaints of sexually active women are reduced lubrication and dyspareunia, the prevalence of which has been reported to be 90% and 80% respectively. Loss of libido and arousal and per vagina bleeding or spotting during or after intercourse are also frequently reported. Urinary symptoms are considered less frequent with dysuria (29%), urgency and urge incontinence (28%), recurrent urinary tract infections, stress incontinence, and voiding issues being some of the most common manifestations [3,6]. Moreover, other common signs of GSM include decreased moisture (94%), loss of vaginal rugae (78%), vaginal pallor (75%), and decreased elasticity (68%). Finally, pelvic organ prolapse, such as cystocele, rectocele, prolapse of the uterus, or vaginal vault prolapse, is also related to GSM [1,2,7].

The prevalence and severity of the above-mentioned symptoms vary in relation to time passed since menopause, with most of them being more frequent and intense five years after menopause when compared with women closer to premenopausal status (GSM symptoms occur in 84% of women six years after menopause versus one year postmenopausally in 65%) [3,8]. Contrary to the vasomotor symptoms related to menopause, which tend to be milder over time, symptoms of GSM appear to have a greater impact on the quality of life (QOL) of the affected women. This is due to the fact that they rarely resolve spontaneously and, in most cases, deteriorate if left untreated, thereby negatively affecting patients' confidence and intimacy with their partners [9,10]. The impact in the QOL has been shown to be more profound in sexually active women [3].

The diagnosis and evaluation of GSM are clinical and mostly established through a thorough medical history and physical pelvic examination [11]. Women who are peri- or postmenopausal or with other causes of hypoestrogenism should be evaluated for signs and symptoms of urogenital atrophy during routine clinical visits [10,11]. Laboratory testing is not typically required, although cultures or biopsies could be indicated if appearance is non-typical or does not improve after therapy [12]. Some tests to assess the efficacy of therapies such as the vaginal pH and the vaginal maturation index (VMI) are mainly used in research studies and are not considered essential for the diagnosis of GSM. Clinicians should always exclude other causes with similar symptoms and, specifically, ﻿dermatological conditions of the vulva such as lichen sclerosus or planus, eczema, dermatitis, chronic vulvovaginitis, vaginitis and vaginosis, ﻿vulvodynia, ﻿malignancies, and ﻿chronic pelvic pain [13]. Recently, two instruments of measurement properties of patient-reported outcome measures (PROMs) specific for genitourinary symptoms (the Vulvovaginal Symptoms Questionnaire and the Day-to-Day Impact of Vaginal Aging (DIVA) Questionnaire) have been shown to be valid after a thorough assessment. It has been shown these two tools can be efficiently used for the evaluation of GSM symptoms and measurement of their impact on the QOL of patients [14,15].

Pathophysiology and risk factors 

Most of the symptoms of GSM are attributed to the lack of estrogen that characterizes menopause. The anatomical and pathophysiological changes caused by GSM are presented in Table 2. 

**Table 2:** Anatomical and pathophysiological changes caused by GSM GSM: genitourinary syndrome of menopause

Pathophysiology of GSM
Loss of labial and vulval thickness
Decreased collagen, elasticity, and blood flow
Reduced vaginal discharge
Reduced pubic hair, subcutaneous fat of labia majora
Reduced labia minora and hymenal remnants
Decreased vaginal cells glucogen => change vaginal microbiome => increased pH
Decreased pelvic floor strength and control
Dry and thin epithelium
Short and narrow vagina
Prolapse (vaginal vault, pelvic organs, urethral)
Decreased bladder capacity and sensation
Vaginal hypersensitivity or decreased feeling

There are three types of endogenous estrogens (estradiol, estrone, and estriol) with varying ratios as age progresses. Estradiol prevails in premenopausal women, whereas estrone, a less effective form, is the predominant estrogen in menopause, explaining the hypoestrogenic environment in these ages [2,11]. The common embryonic origin during fetal development of the lower urinary tract and external genitalia explains the pathophysiologic effects of the lack of estrogens on these anatomical structures. It has been shown that estrogen receptors (a and b) are present in the vagina, the vestibule of the vulva, urethra, trigone of the bladder, and on autonomic and sensory neurons in the vagina and vulva. While both receptors can be found in premenopausal women, only estrogen-b receptors can be detected in postmenopausal women [12]. Irritation and trauma occurring during sexual intercourse is the result of the hypoestrogenic environment of urogenital tract tissue, and It reflects changes in the thickness of the vaginal epithelium and lamina propria, atrophy of smooth muscles, reduction of vaginal-area blood flow, and loss of tissue elasticity (decreased concentration of collagen, elastin, and hyaluronic acid) [5,6]. Diminution of glycogen concentration in vaginal cells results in an alteration in the vaginal microbiome (fewer lactobacilli) and an increase of vaginal pH [1]. The combined effect of these pathophysiological mechanisms results in changes of the urogenital system, mainly as alterations of the vaginal mucosa (pallor and fragility) and discharge, changes in vaginal microbe consistency, a decrease of pubic hair and subcutaneous fat of labia majora, and loss of size of labia minora and vestibular bulbs. In the lower urinary tract, they affect the capacity and contractile ability of the bladder, the urethral sphincter, and the functionality of pelvic floor muscles [16].

The main risk factors recognized to be associated with GSM besides the menopause itself are the absence of vaginal childbirth, alcohol abuse, bilateral oophorectomy, decreased frequency and sexual abstinence, cigarette smoking, non-menopausal hypoestrogenism, lack of exercise, (premature) ovarian failure, and cancer treatments via pelvic irradiation, chemotherapeutic, and endocrinal agents [4,6,17]. Similarly, ﻿low education levels and chronic diseases, mostly urogynecological pathologies, have also been associated with GSM, while BMI has not been correlated with GSM onset [18].

Therapeutic approach

﻿The primary goal of GSM’s treatment is symptom relief. The available treatment options, besides local and systemic hormonal therapy, include lifestyle changes and non-hormonal treatments (Table 3) [5,19,20]. ﻿

**Table 3:** Treatment options for GSM GSM: genitourinary syndrome of menopause; SERM: selective estrogen receptor modulator; DHEA: dehydroepiandrosterone

Therapeutic modalities of GSM		
Non-hormonal therapy	Lifestyle changes (﻿maintenance of sexual activity, smoking cessation)	
	Vaginal lubricants	
	Vaginal moisturizers	
	Liquid lidocaine compresses ﻿to the vulvar vestibule	
	Microablative fractional CO2 laser	
	Non-ablative photothermal erbium: YAG laser	
	Oral ospemifene (SERM)	
	Nutraceuticals (﻿phytoestrogens)	
	Alternative and complementary therapies	Oral vitamin D
		Vaginal vitamin E
		Probiotics
Hormonal therapy	Estrogen systemic	Orally
		Transdermally (patch or gel)
		Subcutaneously (implant)
		Vaginally
	DHEA	
	Testosterone	

Concerning the conservative approach, a positive link between maintenance of vaginal elasticity and lubricative response to sexual arousal with sexual activity has been demonstrated [21]. Moreover, avoiding any risk factors associated with GSM, such as smoking cessation, can be helpful, since smoking has been linked with an increase in estrogen metabolism leading to vaginal atrophy [19]. Nevertheless, the first-line treatment for GSM is the use of non-hormonal vaginal lubricants and moisturizers [22]. Lubricants are water-, silicone-, or oil-based products that are ﻿not skin absorbed. They act immediately and provide temporary relief from vaginal dryness and pain during sex; hence, they are really helpful for women whose vaginal dryness is an issue only or primarily during intercourse. On the other hand, vaginal moisturizers are applied regularly. They are bioadhesives and can improve coital comfort and increase vaginal moisture. Moisturizers mimic vaginal secretions and lower the pH by altering the fluid content in the vaginal epithelium. However, both these products are appropriate mostly for women with mild to moderate symptoms, and many patients will eventually require further hormonal medications [6,23]. Additionally, the use of liquid lidocaine ﻿compresses to the vulvar vestibule before sexual penetration has been described in ﻿breast cancer survivors with menopausal dyspareunia who should not receive estrogen-based therapy. This has shown promising results since 90% of patients reported ﻿comfortable intercourse [24]. Other complementary therapies such as oral vitamin D, vaginal vitamin E, and probiotics have been proposed as alternative modalities to GSM therapy; however, data on their efficacy are scarce and further studies and trials are required for their validation [25-27]. More recently, phytoestrogens, specifically equol, have been proven ﻿effective in modulating symptoms of menopause. However, due to insufficient research and limited data in the literature, they cannot yet be recommended as an alternative treatment option [28]. 

In more persistent cases, hormonal therapy with estrogen products is generally considered the “gold standard”. The use of hormonal therapy is an option for the treatment of moderate to severe GSM symptoms that are not relieved with conservative methods. It may also be used in conjunction with a conservative approach [2,7]. The hormonal therapy includes estrogen-based products that can be administered through different routes (vaginally, orally, transdermally or subcutaneously), with vaginal tablets or creams showing better outcomes than the others [5]. Estrogens delivered orally, in many cases, do not lead to beneficial effects on the lower genital tract. Locally administered Intravaginal estrogen options (tablets, rings, creams) with low estradiol concentration (≤50 mcg estradiol or 50 mcg estriol) have shown good outcomes in treating GSM symptoms such as dryness, itchiness, vaginal mucosa friability, and dyspareunia [11]. Concerning urinary tract symptoms, improvement has been noticed in many cases of recurrent urinary tract infections and overactive bladder (without any sign of microbial involvement), but not in women with stress incontinence. In cases with a history of estrogen-sensitive tumors, such as breast or endometrial cancer, any hormonal therapy should be used with caution; the risk/benefit ratio needs to be individualized and in coordination with an oncologist [19]. Hormonal therapy risks should be evaluated thoroughly and factors such as age, duration of use, dose, type of treatment, route, histologic type of malignancy, and prior exposure should be considered before the prescription of such regimen in survivors of gynecological cancer [29]. Breast cancer is a hormone-sensitive carcinoma in many cases; hence, systemic hormonal therapy is not usually recommended for women with breast cancer [29,30]. As far as endometrial cancer survivors are concerned, the data, although limited, suggest that hormonal therapy is considered relatively safe in low-risk subtypes (i.e., early-stage, low-grade, or type I), but should not be employed in high-risk types [29,31]. Similarly, in survivors of epithelial ovarian cancer, hormonal treatment could be considered in selected cases, since evidence suggests a neutral effect in survival; but it should be avoided in certain histologic types, such as advanced serous and endometrioid and other estrogen-sensitive tumor types [29-31]. 

Another medication used to treat GSM symptoms is the intravaginal dehydroepiandrosterone (DHEA). Studies have shown that DHEA, increases blood estrogen concentration, thereby leading to the improvement of sexual arousal and the return of lost libido [32]. Intravaginal testosterone has shown positive effects in relieving vaginal atrophy symptoms and decreased libido, but its efficacy in GSM generally remains uncertain [33]. Another available option is an oral selective estrogen receptor (SERM) known as ospemifene. It is used mainly for the treatment of dyspareunia and is employed in cases of women who cannot be treated with conventional and medical products due to estrogen contradictions or those who have not responded to other therapies. However, the efficacy of SERMs as a treatment option for GSM needs to be further investigated [34].

Recently, laser and radiofrequency devices have emerged as alternative treatment modalities for GSM [35]. It is suggested that microablative fractional CO2 ﻿and non-ablative erbium: YAG (Er:YAG) lasers restore the tropism in the lower genitourinary tract with three to five sessions [35,36]. Despite the initial excitement for this new therapeutic approach, it has not been fully implemented in the day-to-day practice to this day, and its routine use is not recommended by some scientific societies [20]. Nonetheless, a recent study has reported that laser intervention with ﻿the intravaginal use of either CO2 or Er:YAG laser-technologies is a safe and potentially effective nonpharmacologic intervention for GSM [37].

## Conclusions

GSM results from the hypoestrogenic state that accompanies menopause, and it affects most of the postmenopausal women. Due to different hormonal and anatomical changes, GSM is characterized by various symptoms such as vaginal dryness, dyspareunia, and dysuria, thereby leading to a drastic impact on the QOL of affected women. Recently, different treatment modalities have emerged to treat the condition's bothersome and life-changing symptoms. First-line treatment consists of non-hormonal therapies such as lifestyle changes, lubricants, and moisturizers, while hormonal therapy with locally administered intravaginal estrogen products is considered the “gold standard’’ in more persistent cases. Newer therapeutic approaches with SERMs or laser technologies can be employed as alternative options, but further research is required to analyze their implementation in day-to-day clinical practice.
